# How Glucosinolates Affect Generalist Lepidopteran Larvae: Growth, Development and Glucosinolate Metabolism

**DOI:** 10.3389/fpls.2017.01995

**Published:** 2017-11-21

**Authors:** Verena Jeschke, Emily E. Kearney, Katharina Schramm, Grit Kunert, Anton Shekhov, Jonathan Gershenzon, Daniel G. Vassão

**Affiliations:** Department of Biochemistry, Max Planck Institute for Chemical Ecology, Jena, Germany

**Keywords:** *Spodoptera littoralis*, Mamestra brassicae, Arabidopsis thaliana, glucoraphanin, glucobrassicin, isothiocyanate, detoxification, Lepidoptera

## Abstract

Multiple lepidopteran larvae feed successfully on plants containing glucosinolates despite the diverse array of toxic and deterrent breakdown products, such as isothiocyanates (ITCs), formed upon plant damage. While much is known about how specialist lepidopterans metabolize and tolerate glucosinolates, there is little information about the metabolic fate of these plant defense compounds in specialized herbivores. Employing ^13^C- and ^14^C-labeled 4-methylsulfinylbutyl glucosinolate (glucoraphanin), we identified and quantified the major detoxification products of glucosinolates and ITCs in selected specialized and generalist larvae. While specialists prevented glucosinolate hydrolysis or diverted hydrolysis to form nitriles, hydrolysis in generalists proceeded to toxic ITCs, of which a portion were conjugated to glutathione. However, a large amount of ITCs remained unmodified, which may have led to the observed negative effects on growth and development. The performance of two generalist-feeding caterpillars, *Spodoptera littoralis* (African cotton leafworm) and *Mamestra brassicae* (cabbage moth) on *Arabidopsis thaliana* Col-0 and various glucosinolate-deficient mutants was investigated from hatching until pupation. We found that glucosinolates negatively affected larval growth and development, but not survival, with aliphatic glucosinolates having stronger effects than indolic glucosinolates, and the combination of the two glucosinolate types being even more detrimental to growth and development. Curiously, last instar larvae grew better on wild type than on non-glucosinolate-containing plant lines, but this could not be attributed to a change in detoxification rate or feeding behavior. Glucosinolates thus appear to be effective defenses against generalist lepidopteran herbivores at least during most stages of larval development. Nevertheless, the reversal of negative effects in the oldest instar is intriguing, and further investigation of this phenomenon may shed light on how generalists adjust their physiology to feed on diets with many different types of plant defense compounds.

## Introduction

In their struggle against herbivores and pathogens, plants rely on a large arsenal of defense metabolites to protect their tissues (Wittstock and Gershenzon, 2002; Hartmann, 2007). Herbivores have evolved an equally extensive suite of behavioral, physiological and molecular mechanisms to circumvent plant defenses (Heckel, 2014; Heidel-Fischer and Vogel, 2015). While many previous studies have quantified the effects of plant chemical defenses on lepidopteran herbivores after short-term feeding, few have explored how development is affected by feeding on chemically well-defended plant material for an extended period. More knowledge about whether long-term feeding on defense compounds influences growth and survival will help clarify the linkages between the presence of chemical defense compounds and the evolution of detoxification mechanisms in herbivores.

**FIGURE 1:**
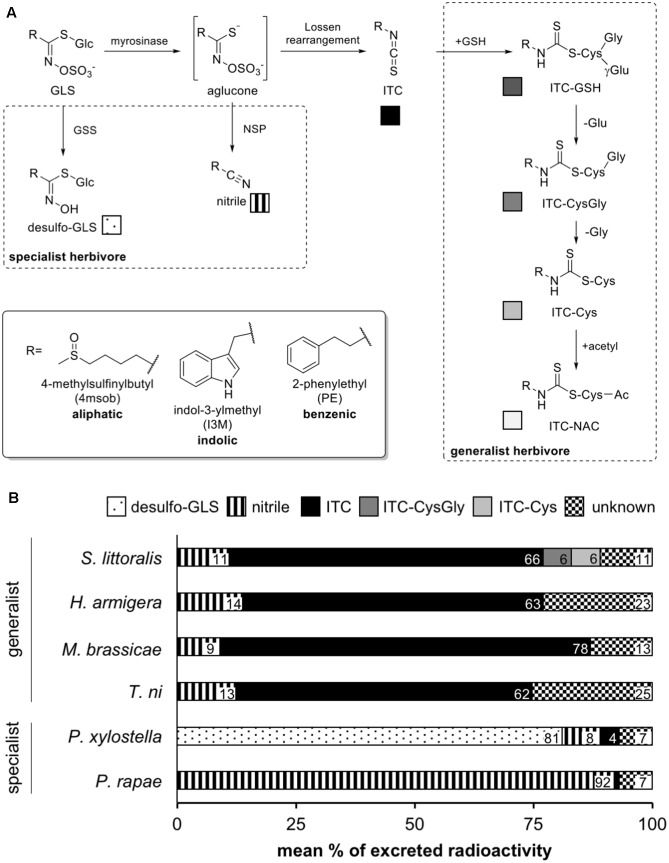
Metabolism of glucosinolates (GLSs) by lepidopteran herbivores. **(A)** In dashed boxes, known pathways of specialist and generalist herbivores are depicted. Specialist adaptations include the production of glucosinolate sulfatases (GSS) to generate desulfo-GLS that cannot be hydrolysed, or the use of nitrile specifier proteins (NSP) to generate nitriles instead of the more toxic isothiocyanates (ITCs). Generalist caterpillars detoxify the ITCs formed upon GLS hydrolysis via conjugation to glutathione (GSH) which is further metabolized via the mercapturic acid pathway. **(B)** The profile of radioactively labeled 4-methylsulfinylbutyl (4msob)-GLS-derived metabolites in the feces of specialist and generalist caterpillars. Intact GLS were not detected. ITC-GSH, -CysGly, -Cys and -NAC: ITC conjugates with glutathione (absent), cysteinylglycine, cysteine and *N*-acetylcysteine (absent), respectively.

Among plant defense compounds, glucosinolates (GLSs) are part of the “mustard oil bomb” of the Brassicaceae and related families, a group that includes agriculturally important crops such as cabbage and rapeseed. GLSs are a chemically diverse group composed of a sulfur- and nitrogen-containing glucosidic core attached to a variable, amino acid-derived side-chain R (**Figure 1A**). They are commonly grouped into three classes based on their precursors: aliphatic, derived from methionine or other aliphatic amino acids; indolic, derived from tryptophan; and benzenic, derived from phenylalanine or tyrosine (Fahey et al., 2001; **Figure 1A**). Intact GLSs are not toxic themselves, but only after their glucose moiety is hydrolyzed by plant myrosinase enzymes (ß-thioglucoside glucohydrolases, EC 3.2.1.147) upon tissue damage resulting in a profusion of potentially toxic products (Wittstock and Burow, 2010). Herbivores specialized on brassicaceous plants detoxify GLSs by well-known mechanisms that prevent GLS hydrolysis or the formation of toxic products (**Figure 1A**; reviewed in Jeschke et al., 2016a). In contrast, generalist herbivores are reported to suffer from GLS hydrolysis and only detoxify the resulting isothiocyanate (ITC) products (Schramm et al., 2012; Zou et al., 2016). However, only few generalist species have been studied and it is not clear what proportion of GLSs are metabolized in this way and whether other modes of detoxification occur in generalist herbivores.

The Brassicaceae model plant, *Arabidopsis thaliana* Col-0, is an ideal organism for GLS studies as it contains a varied assortment of more than 20 different GLSs throughout its development (Brown et al., 2003), and genetically engeneered genotypes are available that do not produce particular classes of GLSs. In the vegetative stage of *A. thaliana* Col-0, the predominant classes in rosette leaves are aliphatic and indolic GLSs, which account for ∼85% and ∼15% of total GLSs, respectively (Brown et al., 2003). Aliphatic GLSs are a large, structurally diverse group in *A. thaliana* with a composition that varies strongly among ecotypes (Kliebenstein et al., 2001). In contrast, indolic glucosinolates consist of a small group of compounds with low structural diversity that is widely distributed in all *A. thaliana* ecotypes. Their biosynthesis and abundance are strongly influenced by environmental factors, such as herbivory (Textor and Gershenzon, 2009). *A. thaliana* Col-0 aliphatic GLSs are hydrolyzed by myrosinase to predominantly form ITCs (Wittstock and Burow, 2010), while indolic GLSs can break down independently of myrosinase activation (Pedras et al., 2002) to form the corresponding nitriles or ITCs, with the latter further decomposing to the carbinol and conjugating to nucleophiles (Agerbirk et al., 2009). In spite of their different properties, the effects of aliphatic vs. indolic GLSs have not always been clearly distinguished, especially for generalist herbivores.

Ingestion of GLSs and their corresponding breakdown products impairs the growth of a wide range of herbivores, and different GLSs have contrasting effects depending on the herbivore studied (reviewed in Jeschke et al., 2016a). Furthermore, quantitative variation of the different GLSs in the host plant significantly affects the insect herbivore diversity and resulting plant fitness (Mithen et al., 1995; Mosleh Arany et al., 2008; Santolamazza-Carbone et al., 2015). As structural and regulatory genes of the GLS biosynthetic pathway have been identified, genetically manipulated plants with altered GLS profiles have become available (Benderoth et al., 2009; Geu-Flores et al., 2009; Sønderby et al., 2010). Short-term feeding studies using such plant lines showed that aliphatic and indolic GLSs have differential effects on the growth of chewing herbivores, with aliphatic GLSs generally having stronger detrimental effects than indolic GLSs (reviewed in Jeschke et al., 2016a). However, how the effects of different GLS classes interact to influence the long-term development of generalist herbivores has not yet been examined.

To study the effects of GLSs on generalist herbivores, we begin by comparing the metabolism of an isotopically labeled GLS in both generalist and specialist lepidopteran caterpillars. The compound fed, 4-methylsulfinylbutyl glucosinolate (4msob-GLS), is the most abundant GLS in the rosette leaves of the Columbia-0 ecotype of *A. thaliana*. Since considerable amounts of free 4msob-ITC were found in the frass of generalists, but not specialists tested, we then investigated the long-term effects of GLS feeding on lepidopteran larvae of two generalist species with different preferences for plants of the Brassicaceae over a long developmental period from neonate to pupation. *Spodoptera littoralis*, the African cotton leafworm, is an extremely polyphagous herbivore infesting plants from 40 different families, including the Brassicaceae (Brown and Dewhurst, 1975). *Mamestra brassicae*, the cabbage moth, is a generalist feeder but one that prefers GLS-containing plants, including several Brassicaceae crop plants (Popova, 1993). Four *A. thaliana* plant lines were used to dissect the effects of aliphatic vs. indolic GLSs in a natural background: (1) Columbia-0 wild type, (2) the double mutant of *cyp79B2* and *cyp79B3* that contains only aliphatic GLSs (Zhao et al., 2002), (3) the double mutant of the *myb28* and *myb29* transcription factors that regulate biosynthesis of aliphatic GLSs, which contains only indolic GLSs (Sønderby et al., 2007; Beekwilder et al., 2008), and (4) the quadruple mutant *myb28myb29cyp79B2cyp79B3* which does not contain detectable levels of either aliphatic or indolic GLSs (Sun et al., 2009). The results give a detailed overview of the effects of GLS feeding on the metabolism, growth and development of generalist lepidopteran insects.

## Materials and Methods

### Plants, Insects, and Chemicals

*Arabidopsis thaliana* was cultivated in a controlled–environment chamber under short-day conditions (10:14 h, light:dark) at 21°C and 50–60% relative humidity (RH). The lines used are the Col-0 wild type, and the following mutants: *cyp79B2 cyp79B3* (aliphatic GLS only) (Zhao et al., 2002), *myb28 myb29* (indolic GLS only) (Sønderby et al., 2007), and *cyp79B2 cyp79B3 myb28 myb29* (no GLS) (Sun et al., 2009). Broccoli (*Brassica oleracea* cv. “Broccocress”) seeds were a generous gift from Discover Freshhh (Monster, The Netherlands), and were germinated and grown under controlled light and temperature conditions (16:8 h, light:dark, day-time temperature 22°C, night-time 20°C). Cabbage plants used for rearing specialist lepidopterans were grown in glasshouses at 22–28°C, with light supplementation as needed for 16:8 h light:dark conditions.

Egg clutches of *Spodoptera littoralis* Boisduval (African cotton leafworm) were a generous gift from Syngenta Crop Protection (Stein, Switzerland), and reared on an artificial diet based on white beans (as described in Jeschke et al., 2016b) at 18–20°C under natural light. Larvae of *Helicoverpa armigera* Hübner (cotton bollworm, Toowoomba strain, generously provided by the Dept. of Entomology, MPI-CE) were reared on a pinto bean diet (Perkins et al., 1973) at 26°C, 16:8 h, light:dark, and 60% RH. Larvae of *Mamestra brassicae* Linnaeus (cabbage moth, provided by the Laboratory of Entomology, Wageningen University of Plant Sciences, The Netherlands) and *Trichoplusia ni* Hübner (cabbage looper, purchased from Benzon Research, Carlisle, PA, United States) were reared on a diet based on wheat germ (Burton, 1969) at room temperature under natural light. Larvae of *Plutella xylostella* (diamondback moth, generously provided by the Dept. of Entomology, MPI-CE) were maintained as in Badenes-Perez et al., 2014. Larvae of *Pieris rapae* (small cabbage white, provided by the Laboratory of Entomology, Wageningen University of Plant Sciences, The Netherlands) were maintained on cabbage plants under short-day conditions (10:14 h, light:dark) at 21°C and 50–60% RH. For the long-term feeding study, larvae were kept at 19°C and under a light cycle of 12:12 h.

Of the chemicals used, 4-methylsulfinylbutyl isothiocyanate (1-isothiocyanato-4-methylsulfinylbutane, sulforaphane), reduced L-glutathione (GSH), D-(+)-glucose, D-(-)-fructose and albumin from bovine serum (BSA) were obtained from Sigma–Aldrich (Munich, Germany). D-(+)-sucrose, Tris, acetonitrile, chloroform, and formic acid (LC grade) were obtained from Roth (Karlsruhe, Germany). Bradford reagent was obtained from Serva (Heidelberg, Germany) and the^13^C/^15^N labeled amino acid standard mix was obtained from Isotec (Miamisburg, OH, United States). Conjugates of 4msob-ITC (4msob-GSH, 4msob-Cys and 4msob-NAC) were purchased from Santa Cruz Biotechnology, Inc. (Dallas, TX, United States). 4msob-CysGly was synthesized as described in (Schramm et al., 2012). Methanol was purchased from Merck (Darmstadt, Germany). All chemicals were obtained in the highest available grade and solvents were in the analytical grade.

### Administration of Isotopically Labeled GLSs to Lepidopteran Larvae

The production of isotopically labeled [^13^C]- and [^14^C]-4msob-GLS, introduction into detached leaves of the *A. thaliana myb28myb29* double knock-out mutant, feeding set-up, feces collection, extraction and HPLC analysis were performed as described in Schramm et al. (2012). The remaining solid feces material was then additionally extracted with Me_2_CO and hexanes sequentially to check for the presence of less polar radioactive compounds via scintillation counting. An aliquot of the final solid residue, as well as crushed insect tissues, were also analyzed by scintillation counting for quantification of radioactivity. ^13^C-Labeled and unlabeled metabolites were identified by LC-MS as previously described (Schramm et al., 2012; Jeschke et al., 2016b; Malka et al., 2016).

### Long-Term Feeding of *S. littoralis* and *M. brassicae* on *A. thaliana* of Varying GLS Content

Larvae were offered leaves of a single line of *A. thaliana* under no-choice conditions starting from when they were newly hatched. Larvae were fed *ad libitum* on leaves detached daily from 6 to 7 week-old plants at the pre-bolting stage. The experiment was run in two different phases: (1) early development (from hatching until 3rd instar) in which larvae were measured (weights, instar changes, and feces collections) in groups of ten, and (2) late development (3rd instar until pupation) in which larvae were measured as individuals. Larvae from different feeding groups were reared concurrently, and the placement of the Solo^®^ cups among trays was randomized daily. Fresh larval weights were recorded in mg with two decimal digits with a Mettler-Toledo XP26 microbalance (Giessen, Germany), at approximately the same time each morning. *Early development:* Neonate larvae of *S. littoralis* and *M. brassicae* were placed in groups of 10 into Solo^®^ cups that were lined with moist filter paper. The cups were then randomly assigned to plant lines with ten replicates per line. Larvae were counted, weighed and checked for instar change every day until all larvae reached the 3rd instar. *Late development:* Neonate larvae of both species were separated in small Solo^®^ cups in random groups of 40, with six replicates per plant line. They were fed exclusively with the designated plant line, but no data were collected until the later stages of development. With the onset of the 3rd instar (on average on the 7th day for *S. littoralis* and the 10th day *M. brassicae*), three larvae out of each of the six group cups were each transferred singly into an individual Solo^®^ cup for a total number of 18 larvae per plant line. This marked the start of the individual phase. The weight of each larvae was recorded every second day and the instar of the larvae was recorded daily. Two days after molting into the 6th instar, potting soil was added to the cups to provide a suitable substrate in which the larvae could pupate. Date of pupation was recorded as the day in which the larva was no longer visible on top of the soil.

The feces were collected from the cups starting on day 3 and thereafter every second day into Eppendorf tubes for metabolite analysis. Feces preparation, extraction and metabolite analysis were carried out as described in Jeschke et al. (2016b).

### Chemical Analysis of *A. thaliana* Lines of Varying GLS Content

Whole rosettes of all four Arabidopsis lines were collected thrice weekly for plant quality control. GLSs were analyzed with *p*-hydroxybenzyl GLS (sinalbin) as internal standard as described in Schramm et al., 2012. The contents of protein, sugar, and amino acids were determined by extracting 10 mg freeze-dried ground plant material in 100 μL aqueous buffer (Tris, 50 mM, pH 7.5). Protein and sugar were determined as described in Jeschke et al. (2016b). Amino acids were analyzed as described in Docimo et al. (2012).

### Determination of Leaf Fragment Sizes in Insect Feces

*Spodoptera littoralis* larvae of the 4th and 6th instars were allowed to feed *ad libitum* for 24 h on detached leaves of *A. thaliana* WT or the no-GLS line (*N* = 5). The feces were collected, resuspended in 1 mL H_2_O and three drops of the suspension were plated on individual microscope slides. Using a Zeiss Axiovert 200 microscope connected to an AxioCam MRc 5 digital camera, we took five pictures of different areas of the slides and determined the perimeter of all fragments in each area using Adobe Photoshop^®^. Pictures derived from one caterpillar were treated as technical replicates in the analysis and averaged.

### Statistical Analysis

All statistical testing was performed using the software R 3.0.2 (R Core Team, 2017) unless otherwise noted. Data are presented as mean ± standard error. Data were controlled for statistical prerequisites such as homogeneity of variances and normality. For differences in survival of the young larvae within the first seven days, Kaplan–Meier curves were generated using a right-censored regression analysis (R survival package) (Therneau, 2015). Models with various distributions were conducted and compared with an analysis of deviance to find the model with the best fit. In case of significant difference a Tukey test (multicomp package, Hothorn et al., 2008) was performed. Weight data was analyzed using generalized least squares models (R nlme package (Pinheiro et al., 2017); general formula: Weight ∼ Treatment). To account for the variance heterogeneity of the residuals between feeding treatments (except for weight gain in the fifth instar of *S. littoralis*) the varIdent variance structure was used to allow the variance of the residuals of each feeding treatment to vary independently. Pairwise comparisons between treatments were performed using least squares means with the lsmeans package (Lenth, 2016) with a Tukey HSD adjustment of the *p*-values. The average day of an instar change was determined by plotting the % of caterpillars that had changed into the new instar each day. A sigmoidal curve (Boltzmann function) was then fitted and the inflection point (x_0_) represents the day at which 50% of caterpillars had reached the new instar. The fitting of the sigmoidal function and calculation of the inflection point (with standard error) was performed in Origin 8 SR2 (v8.0891, OriginLab Corporation, United States) with further statistical analysis (ANOVA and Tukey *post hoc* test) using SigmaPlot 11 (v11.2.0.5, Systat Software, Inc.). Larval weight gain per instar was calculated as the difference of the weight at the onset of two sequential instars. Statistical testing was performed with ANOVA. In case of inhomogeneity of variance (Fligner–Kileen test, *P* < 0.05) a Kruskal–Wallis test was used. Percentages of survival to day 6 were calculated per cup (survival of original 40 neonate larvae) and the percentages of 10 cups per treatment were averaged. Statistical testing was performed with an ANOVA. Percentages of survival to 6th instar and pupation were compared with a test of proportions (prop.test). The ITC detoxification products in the feces were analyzed for the early developmental phase (1st and 2nd instar) per days and for the later developmental phase (3rd to 6th instar) per instar. In cases where several feces collections per larva were made for a certain larval stage, the mean of these measurements was used in the analyses. Detoxification products of ITC with GSH were analyzed with linear mixed models with the lme function (nlme package, Pinheiro et al., 2017) to account for the repeated measurement of feces from groups or individual larvae. Treatment and day (early phase) or instar (later phase) were treated as fixed effects. Larval group or individual larvae were treated as random effects. The data were transformed to log values to fulfill the requirements for the test. *P*-values for explanatory variables were obtained by deleting explanatory variables one after another and comparison of the likelihood of the more complex model with that of the simpler model (Zuur et al., 2009). Factor level reductions were used to reveal differences between different larval stages (Crawley, 2013).

## Results

### Generalist Lepidopteran Herbivores Metabolized GLSs to Free ITCs and Glutathione Conjugates

To investigate the effect of glucosinolates (GLSs) on four species of generalist feeding lepidopteran larvae, we first compared their GLS metabolism by determining the metabolic fate of a dose of [^13^C]- and [^14^C]- 4-methylsulfinylbutyl glucosinolate (4msob-GLS, glucoraphanin). The ^13^C label allowed identification of metabolites by LC-MS when necessary, while the ^14^C label permitted quantitative comparison of the flux into various metabolites by direct radioactivity measurement. The four lepidopteran species studied included two showing a high natural preference for GLS-containing plants (*Mamestra brassicae* and *Trichoplusia ni*) (Popova, 1993; Chow et al., 2005) and two with little or no preference (*Spodoptera littoralis* and *Helicoverpa armigera*) (Brown and Dewhurst, 1975; Firempong and Zalucki, 1989). For contrast, we also included measurements of the GLS metabolism of two specialist lepidopterans, *Pieris rapae* and *Plutella xylostella*, whose major GLS metabolites have been described (Ratzka et al., 2002; Wittstock et al., 2004).

For all species, nearly 100% of the ingested radioactivity was recovered in the final extracts (**Figure 1B**). For the specialists, the large majority of ingested GLSs were excreted as the nitrile for *P. rapae* and as the desulfo-GLS for *P. xylostella*, consistent with previous reports (Ratzka et al., 2002; Wittstock et al., 2004). However, in generalist species the major share of ingested 4msob-GLS (62–78%) was excreted as the free isothiocyanate (ITC), while evidence of ITC conjugation with glutathione was detected for *S. littoralis* here and in the long-term feeding experiment for *M. brassicae* (see below). Approximately 10% of the ingested GLS in each generalist species was excreted as the corresponding nitrile. Since this proportion is similar to that found in the hydrolysis products formed by *A. thaliana* leaves infused with [^14^C]-4msob-GLS, then crushed and extracted, it may not be a consequence of insect metabolism. The remainder of the ingested GLS (up to 25%) was excreted as unidentified, very polar compound(s) with no retention on the reverse phase HPLC column used (Schramm et al., 2012).

In conclusion, all generalist species excreted large amounts of unconjugated ITCs, and thus there was no relation between their GLS metabolism and their degree of feeding preference for GLS-containing plants. The large amount of free ITCs released suggested that GLSs would have negative impacts on these insects. Hence long-term feeding studies were undertaken with one generalist with no preference for GLS-containing plants (*S. littoralis*) and one with a decided preference (*M. brassicae*) to investigate how GLSs affect insect survival, growth and development.

### Mutant Lines with Altered GLS Content Did Not Differ from Wild Type in Levels of Most Sugars, Proteins, and Amino Acids

**FIGURE 2:**
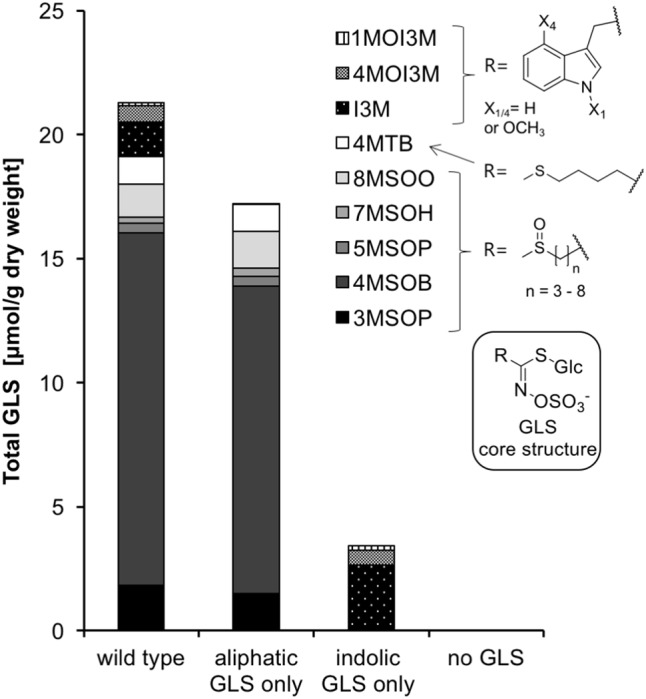
The GLS content of 7-week-old plants of *A. thaliana* Col-0 (wild type) and the biosynthetic knock-down mutants *cyp79B2cyp79B3* (aliphatic GLS only), *myb28myb29* (indolic GLS only) and *cyp79B2/3myb28/29* (no GLS). For the exact values as well as sugar, soluble protein and amino acid content, refer to Supplementary Table S1. Abbreviations for GLSs: 1MOI3M/4MOI3M: 1- and 4-methoxyindol-3-ylmethyl, I3M: indol-3-ylmethyl, 4MTB: 4-methylthiobutyl, 8MSOO: 8-methylsulfinyloctyl, 7MSOH: 7-methylsulfinylheptyl, 5MSOP: 5-methylsulfinylpentyl, 4MSOB: 4-methylsulfinylbutyl, 3MSOP: 3-methylsulfinylpropyl.

Four *A. thaliana* lines were used to examine the effects of GLSs on caterpillar development: (1) Columbia-0 wild type (WT) containing native levels of aliphatic and indolic GLSs, (2) the double mutant of *cyp79B2 cyp79B3* that contains only aliphatic GLSs and is devoid of indolic GLSs (Zhao et al., 2002), (3) the double mutant of the *myb28 myb29* transcription factors that regulate biosynthesis of aliphatic GLS, which contains only indolic and no aliphatic GLSs (Sønderby et al., 2007; Beekwilder et al., 2008), and (4) the quadruple mutant *myb28/29 cyp79B2/B3* with undetectable levels of aliphatic and indolic GLS (Sun et al., 2009). WT Col-0 rosette leaves (6–7 weeks old) contained on average 21.29 ± 2.31 μmol/g dry weight total GLSs (**Figure 2** and Supplementary Table S1). Aliphatic GLS accounted for ∼90% of the total, and were present in similar concentrations in leaves of *cyp79B2cyp79B3* (aliphatic GLS only line) as in WT Col-0. Indolic GLS (∼10% of the total in Col-0 WT) were 1.6-fold more concentrated in leaves of *myb28myb29* (indolic GLS only line) than in Col-0 WT. The quadruple mutant *myb28/29cyp79B2/3* (no GLS line) accumulated only trace levels of GLS. Additionally, we looked for differences among the lines in other metabolites that could influence insect feeding, including soluble protein, free proteinogenic amino acids and simple sugars (sucrose, fructose and glucose + galactose) (Supplementary Table S1). There were no significant differences among the four lines except for an elevated glucose + galactose content in the aliphatic GLS only line.

### GLSs Reduced Larval Growth Rate

The development of *S. littoralis* and *M. brassicae* larvae on the four lines of *A. thaliana* Col-0 was observed from hatching until pupation to compare the influence of different GLS classes. Developmental stages were grouped as: (1) early larval development, from hatching to the beginning of the 3rd instar, and (2) late larval development, from 3rd instar to pupation. We assessed mortality, growth rate, and the duration of development.

**FIGURE 3:**
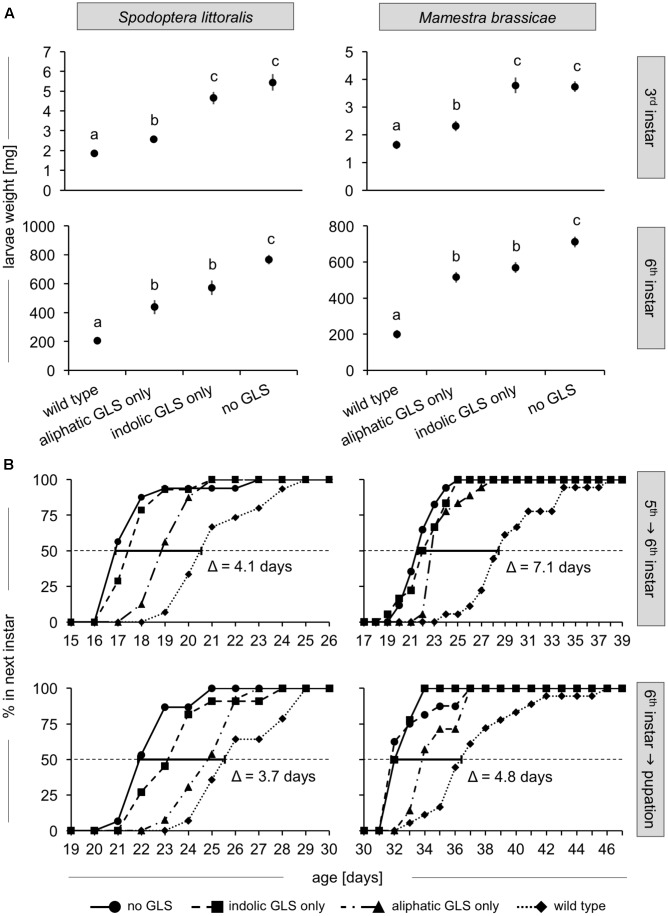
Growth and development of *S. littoralis* and *M. brassicae* larvae on GLSs. **(A)** Larval fresh weights at the onset of the 3rd instar (upper panels, day 6 for *S. littoralis, P* < 0.001, *N* = 10; day 10 for *M. brassicae*; *P* < 0.001, *N* = 10), and entering 6th instar (lower panels, day 20 for *S. littoralis, P <* 0.001, *N* = 13–17; day 28 for *M. brassicae, P* < 0.001, *N* = 17–18). Data points represent the estimates of the means ± standard error from the gls model (Supplementary Table S3). Statistical analysis was performed using an *ANOVA*, and the letters denote significantly different groups based on a *Tukey post hoc test* (0.05 level). **(B)** Instar transition time for 5th to 6th instar and for 6th instar to the pupal stage. The dotted horizontal line indicates when 50% of the caterpillars had reached the next developmental stage. The bold line shows the time intervals between the transition times for larvae fed on WT and no-GLS plant lines calculated by fitting a sigmoidal curve to the data (see Materials and Methods). Data for the mean day ± standard error, and *ANOVA* and *Tukey post hoc* analysis for all instars are in Supplementary Table S4.

No significant treatment-related effects were observed in either species on mortality during early and late larval development stages and on pupation success (Supplementary Table S2). However, the different food plants significantly influenced larval growth during both early and late development in both species (**Figure 3A**, Supplementary Figure S1 and Supplementary Table S3). By 6 days after hatching (at the end of the early development phase), larvae reared on plants containing no GLSs or only indolic GLSs had grown equally well. In contrast, larvae fed on plants containing only aliphatic GLS exhibited significantly reduced growth reductions of 53 and 38% for *S. littoralis* and *M. brassicae*, respectively, compared to larvae fed on the plant line with no GLSs. This reduction was even stronger for larvae fed on WT Col-0 (66 and 56% for *S. littoralis* and *M. brassicae*, respectively, compared to those fed on the no-GLS line). By the onset of the 6th instar (day 20 for *S. littoralis* and day 28 for *M. brassicae* fed on no GLS-plants), larvae fed on plant lines with indolic GLS only also showed a significant reduction in growth (25 and 20% for *S. littoralis* and *M. brassicae*, respectively) compared to larvae fed on plants with no GLSs, while larvae fed on aliphatic GLS-only plants showed an even stronger reduction (43 and 27% for *S. littoralis* and *M. brassicae*, respectively). Again, larvae reared on WT Col-0 had significantly lower weights than those fed on any of the other plant lines (73 and 72% reduction *S. littoralis* and *M. brassicae*, respectively) compared to larvae fed on plants without GLSs.

### GLSs Extended Larval Development Time

To examine whether feeding on plants of different GLS content influenced the duration of larval instars, the time points of instar change were defined as the day on which 50% of caterpillars in a feeding treatment entered the next instar (dotted line, **Figure 3B**). Instar duration in both species was lengthened by the ingestion of GLS. Aliphatic GLS had a more pronounced effect than indolic GLS, and their combination in WT Col-0 caused an even stronger effect. First instar duration was already lengthened in WT Col-0-fed compared to no-GLS-fed larvae by 1.4 and 0.9 days for *S. littoralis* and *M. brassicae*, respectively (Supplementary Table S4). For each following instar change, the WT Col-0-fed caterpillars experienced an additional delay compared to caterpillars fed on no-GLS plants. By the last instar change, this resulted in an accumulated delay of 4.1 days for *S. littoralis* and 7.1 days for *M. brassicae* between the WT-fed and no GLS-fed treatments.

Surprisingly, the cumulative developmental time difference between wild type and no-GLS-fed caterpillars decreased at the time of pupation. That is, WT-fed insects “caught up” during their last instar, reducing the gap between groups: treatment-associated gaps measured during molting into the last larval instar were shortened by 0.4 days for *S. littoralis* and 2.3 days for *M. brassicae* on pupation. A comparison of the growth rates in each instar during late development stages was therefore performed (**Table 1**). The growth rates (mg/instar) were 48 and 42% higher for the no-GLS-fed larvae than those fed WT Col-0 in the 3rd and 4th instar for *S. littoralis* and *M. brassicae*, respectively. However, this pattern was reversed during the 6th instar, when larvae fed on WT Col-0 leaves grew significantly faster (by 1.4-fold for both species) than larvae fed on no-GLS plants. Examination of pre-molt larval weights (Supplementary Table S5) showed that the average weight before the 4th instar in *M. brassicae* reflects the trends observed during development: Larvae fed on wild type plants are significantly lighter than larvae that were challenged with only one GLS class or none (Supplementary Figure S1). The weights before the last instar change did not significantly differ between treatments. Similarly, the average weight before each molt did not differ between treatments for *S. littoralis* larvae. However, *S. littoralis* larvae fed on GLS-containing plants showed a trend in the 5th and 6th instar toward higher pre-molting weight than no GLS-fed larvae. Both of these developments reflect the trends in growth rate in the later instars.

**Table 1:** Contrasting effect of feeding on various GLS-containing plant lines on larval growth at different stages of development.

Instar		Plant line fed on					
		Wild type	Aliphatic GLS only	Indolic GLS only	No GLS	*P*-value	*F/χ^2#^* value
*Spodoptera littoralis*	3rd	3.20 ± 0.26 (a)	4.17 ± 0.34 (ab)	4.12 ± 0.42 (ab)	**4.74 ± 0.42 (b)**	0.030	3.203
	4th	18.79 ± 1.20	18.73 ± 1.81	17.96 ± 2.96	16.16 ± 1.13	n.s.^#^	1.941
	5th	69.80 ± 5.55	68.62 ± 5.24	69.06 ± 7.33	83.95 ± 5.66	n.s.	1.653
	6th	**212.05 ± 20.87 (a)**	135.88 ± 11.20 (b)	114.83 ± 8.40 (b)	146.72 ± 9.27 (b)	<0.001	8.985
*Mamestra brassicae*	4th	6.55 ± 0.67 (a)	8.86 ± 0.64 (ab)	8.16 ± 0.61 (ab)	**9.33 ± 0.67 (b)**	0.026	3.291
	5th	33.65 ± 2.88	34.53 ± 1.45	33.37 ± 1.61	30.51 ± 1.46	n.s.	0.874
	6th	**94.59 ± 3.73 (a)**	56.19 ± 2.41 (b)	75.22 ± 3.91 (c)	68.54 ± 7.71 (c)	<0.001^#^	35.070


*The larval mass gained for each instar (in mg) is presented. Listed are the means ± standard error (*N* = 10–16 for *S. littoralis*, *N* = 14–18 for *M. brassicae*). The bold print highlights the plant treatment giving the highest mass gain when these were significantly different. Statistical analysis was performed using *ANOVA* or ^*#*^*Kruskal–Wallis test* and the letters denote significantly different groups based on a *Tukey post-hoc test* (0.05 level; see Materials and Methods for details)*.

### ITC Detoxification Rate, But Not Leaf Fragment Size Changed during Larval Development

To try to account for the changing growth rates of caterpillars on different plant lines during development, we quantified the detoxification products found in the feces at different developmental stages from the major aliphatic GLS, 4msob-GLS (**Figure 2**), which is hydrolyzed to 4msob-ITC. Both *S. littoralis* and *M. brassicae* detoxified 4-msob-ITC by conjugation to GSH and then metabolized the initial conjugate further via the mercapturic acid pathway (**Figure 1A**). We measured the feces of larvae that had fed on plants containing 4msob-GLS, i.e., those fed on WT plants and those fed on plants with aliphatic GLS only (**Figure 3**). The total amounts of detoxification products excreted by *S. littoralis* were higher than those found in the feces of *M. brassicae* and depended significantly on the stage of larval development, decreasing with age during both early and late development phases. In contrast, *M. brassicae* larvae showed a trend toward increased detoxification during development with a significant increase at day 10 (Supplementary Table S6). The relative distribution of detoxification products changed over time for both species, with increasing proportions of later products of the mercapturic acid pathway (NAC- and Cys-conjugates) instead of earlier ones (CysGly- and GSH- conjugates). The presence of indolic GLS (in WT Col-0) did not significantly influence the total amount of these 4msob-ITC detoxification products in either species, with larvae reared on plants with aliphatic GLS only not excreting more detoxification products than larvae reared on WT Col-0 (Supplementary Tables S6, S7).

Another way to account for changing growth or detoxification rates of larvae during development would be if insects changed their feeding behavior. Lepidopteran larvae feeding on cyanogenic glucoside-containing plants have been reported to snip off relatively large leaf fragments and so cause less tissue damage and reduced formation of toxic hydrolysis products (Pentzold et al., 2014). Production of larger leaf fragments might results from larger mandible size or a change in feeding behavior. We hypothesized that larger *S. littoralis* larvae might encounter fewer hydrolysis products than smaller larvae due to the decrease observed in metabolites of 4msob-ITC in frass. However, the size of leaf fragments in the feces of 4th and 6th instar *S. littoralis* caterpillars fed on WT plants did not differ from those fed on no-GLS plants (Supplementary Table S8). Neither instar nor plant treatment influenced the average leaf fragment sizes in frass.

## Discussion

### GLS Metabolism by Generalist Lepidopterans Is Completely Different from That of Specialists

Despite the potential for GLSs to be hydrolyzed on plant damage and form toxic products, many insects feed and perform well on GLS-containing species. This has been attributed to several well-described detoxification processes (reviewed in Jeschke et al., 2016a). For instance, among specialist lepidopteran feeders on GLS-containing plants, *P. rapae* diverts GLS hydrolysis to form nitriles instead of the more toxic ITCs (Wittstock et al., 2004), while *P. xylostella* modifies GLSs by desulfation, which prevents myrosinase-catalyzed hydrolysis altogether (Ratzka et al., 2002), but information is lacking on other possible routes of GLS metabolism in these insects. Five species of generalist feeding lepidopterans have been reported to deactivate ITCs by conjugation to GSH (Schramm et al., 2012), a general detoxification pathway for nucleophilic toxins in many organisms, but most of these insects have not been systematically analyzed for other potential GLS metabolites. The feeding of radiolabeled 4msob-GLS in this study to both specialists and generalists has now put their GLS metabolism on a firmer quantitative basis.

Specialist lepidopteran herbivores were found to metabolize 4msob-GLSs principally to specialized detoxification products, including over 80% conversion to desulfo-GLSs in *P. xylostella* and over 90% conversion to nitriles in *P. rapae*, with no evidence of ITC conjugation. On the other hand, metabolism in the generalist lepidopterans studied gave rise principally to GSH conjugates of 4msob-ITC or free 4msob-ITC (60–80%) without any detection of desulfo-GLSs (**Figures 1**, **3**). A background level of 8–15% nitrile formation was found in all feeding experiments, probably a consequence of *in planta* nitrile formation after activation of labeled glucosinolates in this experiment. Thus there are fundamental differences in the way that specialist and generalist lepidopteran larvae process dietary glucosinolates. Among the four generalists investigated, there were no differences in the gross patterns of GLS metabolism despite the fact that their frequency of feeding on GLS-containing plants varies. *M. brassicae* and *T. ni* use Brassicaceae frequently as host plants, while *S. littoralis* and *H. armigera* are seldom reported on GLS-containing plants (Brown and Dewhurst, 1975; Firempong and Zalucki, 1989; Popova, 1993; Chow et al., 2005). The relatively high proportion of free ITCs observed in the feces of generalists (up to 78%) was surprising. The detoxification of ITCs with via conjugation to GSH may not be very efficient, or alternatively these toxic GLS hydrolysis products could have arisen from ITCs initially conjugated with GSH but then dissociated. This process could reduce the nitrogen and sulfur cost of conjugation by recovering cysteine and other amino acids but could also increase the risk of toxicity.

### GLSs, Especially Aliphatic GLSs, Reduce Generalist Lepidopteran Performance over Much of the Larval Life Span

The abundance of free ITCs as GLS metabolites in generalist lepidopterans motivated us to explore GLS effects on larval performance in detail. While most previous studies of this type have been of short duration (reviewed in Jeschke et al., 2016a), we investigated larval survival, growth and development from neonate to pupation employing *A. thaliana* lines knocked out in aliphatic or indolic GLS biosynthesis, the same lines previously used in many herbivore feeding studies (Zhao et al., 2002; Beekwilder et al., 2008; Sun et al., 2009). Chemical analysis of these plant lines confirmed the GLS composition previously reported. In addition, we found no major differences among the lines in sugar, protein or free amino acid content making it likely that GLS are responsible for any differences in insect performance observed.

The presence of aliphatic or indolic GLSs or both significantly reduced the larval growth rate of the two lepidopteran species studied (**Figure 3A**) leading to longer development times and a delay in pupation (**Figure 3B**, Supplementary Figure S1 and Supplementary Table S4). The effect of both GLS classes together was stronger than either one alone. Both classes of GLSs have been previously demonstrated to reduce the growth of generalist lepidopteran larvae (Beekwilder et al., 2008; Schlaeppi et al., 2008; Müller et al., 2010). The longer larval development could be detrimental in a natural setting due to longer exposure to predators (the slow growth-high mortality hypothesis) and may result in other fitness costs such as fewer generations per season (Clancy and Price, 1987; Benrey and Denno, 1997). In fact, *S. littoralis* larvae raised on cabbage plants gave rise to adult moths that avoided Brassicaceae as host plants for oviposition (Thöming et al., 2013; Proffit et al., 2015) demonstrating that – presumably negative – larval experiences on brassicaceous plants later influence adult choices. In contrast to the effects on growth and development, the presence of GLSs had no effect on larval survival. However, some previous studies have indeed found higher mortality of lepidopteran larvae when fed on GLS-containing diets (Lichtenstein et al., 1962; Li et al., 2000; Agrawal and Kurashige, 2003; Ahuja et al., 2010; Santolamazza-Carbone et al., 2015).

**FIGURE 4:**
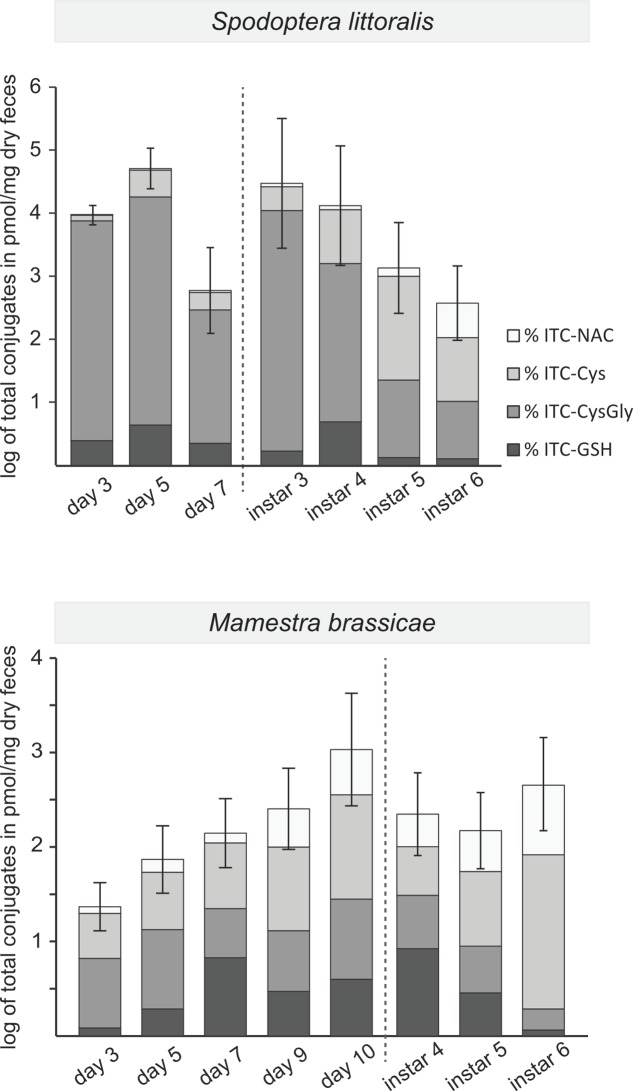
Excretion of 4msob-ITC detoxification products in the feces at different stages of larval development. The sum of the ITC conjugates is plotted in log scale (mean ± standard error) with a division (dotted line) between early development (1st to 3rd instar, listed by day) and late development (listed by instar). Relative amounts of the individual metabolites within each bar are shaded in gray tones (expressed as percentages of the total). The total amounts of conjugates and percentages are averaged for larvae fed on the two plant lines containing aliphatic GLS (WT Col-0 and aliphatic GLS only). *S. littoralis*, early development: factor (day): *P* = 0.003, late development: factor (day): *P* < 0.001; *M. brassicae*, early development: factor (day): *P* = 0.020, late development: factor (day): *P* n.s (see Supplementary Table S6 for detailed information).

In general, aliphatic GLSs seem to be more detrimental to lepidopteran larvae performance than indolic GLSs (Schlaeppi et al., 2008; Müller et al., 2010; Jeschke et al., 2016a), as found in this study also. The weight reduction caused by feeding on WT Col-0 was greater than that of either GLS class alone, suggesting synergistic or additive effects between two classes as previously proposed for *Spodoptera exigua* (Müller et al., 2010). However, synergism cannot be rigorously tested here since the indolic GLS-only plant line contained significantly more of this class than the WT line (**Figure 2**). Nevertheless, we can exclude the possibility that the presence of indolic GLS inhibits the detoxification of aliphatic GLS and vice versa (**Figure 4**).

### Older Larvae May Grow Better on GLS-Containing vs. Non-GLS Plant Lines

Despite the overall negative impact of GLSs on the growth of both lepidopteran species during larval development, GLS-fed insects seemed paradoxically to “catch up” to non-GLS-fed controls during the last instar. For example, *S. littoralis* larvae fed on WT plants grew significantly faster in their final instar than those fed on the plant line without GLSs (**Table 1**). There are several possible explanations for this unexpected reversal of GLS impact in older larvae. One is that a change in the direction of GLS metabolism occurred allowing older insects to degrade GLSs and exploit the nitrogen and sulfur for their own growth and development. However, no major changes in the course of metabolism were evident between the penultimate and ultimate instars (**Figure 4**).

Another possible explanation for the improved ability of older larvae to grow on GLS-containing diets is that the rate of detoxification increased with age. This is true in *M. brassicae* at some early stages in development, perhaps because of increases in the efficiency of ITC conjugation due to more GSH or higher amounts of glutathione *S*-transferase activity. However, in *S. littoralis*, the rate of GLS detoxification actually declined with development. Less ITC might be reaching the midgut epithelial tissue in older larvae due to the larger volume of the gut, or insects retain the food bolus for a smaller timespan. Alternatively, previously conjugated ITCs may undergo more complete dissociation to free ITCs in older larvae resulting in fewer conjugates being excreted. This would benefit larvae by salvaging cysteine residues, which are limiting for GSH biosynthesis (Jeschke et al., 2016b). Or, older larvae may bite off and ingest larger leaf fragments due to their larger mandibles and thus cause less cell damage and subsequently less GLS hydrolysis. Feeding behavior that gives larger leaf fragments has been observed during lepidopteran herbivory on cyanogenic glucoside-containing plants, and reduces exposure to toxic hydrogen cyanide (Pentzold et al., 2014; Pentzold et al., 2015). However, we found no evidence that *S. littoralis* increases the size of the leaf fragments it produces in the last instar or as a result of increased GLSs in the diet (Supplementary Table S8).

## Conclusion

The metabolism of GLSs by the generalist lepidopterans studied does not block the activation of these defenses, but instead conjugates toxic ITCs to glutathione after their hydrolysis. This metabolism prevents mortality, but does not stop GLSs from decreasing the growth and development of these insects. Thus GLSs may still function as effective defenses against generalist lepidopteran herbivores if longer development times increase the risk of predation. In addition, these compounds may act defensively as feeding deterrents, a property not tested here. Sub-lethal effects may be more typical for plant defenses than outright lethality in part because synthesis of concentrations high enough to kill feeding herbivores may be too expensive considering the actual risks of herbivory. Growth reduction, deterrency and increased predation may together be sufficient to reduce herbivore levels.

## Author Contributions

VJ, EK, JG, and DV conceived and designed the experiments. EK, KS, AS, and DV performed the metabolism experiment. VJ and EK performed the development experiment. VJ, EK, and GK performed statistical analysis and VJ, EK, JG, and DV interpreted the results and wrote the manuscript.

## Conflict of Interest Statement

The authors declare that the research was conducted in the absence of any commercial or financial relationships that could be construed as a potential conflict of interest. The reviewer TG and handling Editor declared their shared affiliation.

## References

[B1] Agerbirk N. Vos M. Kim J. H. Jander G. (2009). Indole glucosinolate breakdown and its biological effects. *Phytochem. Rev.* 8 101–120. 10.1007/s11101-008-9098-0

[B2] Agrawal A. A. Kurashige N. S. (2003). A role for isothiocyanates in plant resistance againts the specialist herbivore *Pieris rapae*. *J. Chem. Ecol.* 29 1403–1415. 10.1023/A:1024265420375 12918924

[B3] Ahuja I. Rohloff J. Bones A. M. (2010). Defence mechanisms of Brassicaceae: Implications for plant-insect interactions and potential for integrated pest management. A review. *Agron. Sustain. Dev.* 30 311–348. 10.1051/Agro/2009025

[B4] Badenes-Perez F. R. Gershenzon J. Heckel D. G. (2014). Insect attraction versus plant defense: young leaves high in glucosinolates stimulate oviposition by a specialist herbivore despite poor larval survival due to high saponin content. *PLOS ONE* 9:e95766. 10.1371/journal.pone.0095766 24752069 PMC3994119

[B5] Beekwilder J. van Leeuwen W. van Dam N. M. Bertossi M. Grandi V. Mizzi L. (2008). The impact of absence of aliphatic glucosinolates on insect herbivory in Arabidopsis. *PLOS ONE* 3:e2068. 10.1371/journal.pone.0002068 18446225 PMC2323576

[B6] Benderoth M. Pfalz M. Kroymann J. (2009). Methylthioalkylmalate synthases: Genetics, ecology and evolution. *Phytochem. Rev.* 8 255–268. 10.1007/s11101-008-9097-1 16754868

[B7] Benrey B. Denno R. F. (1997). The slow-growth-high-mortality hypothesis: a test using the cabbage butterfly. *Ecology* 78 987–999. 10.1890/0012-9658

[B8] Brown E. S. Dewhurst C. F. (1975). Genus *Spodoptera* (Lepidoptera, Noctuidae) in Africa and near East. *Bull. Entomol. Res.* 65 221–262. 10.1017/S0007485300005939

[B9] Brown P. D. Tokuhisa J. G. Reichelt M. Gershenzon J. (2003). Variation of glucosinolate accumulation among different organs and developmental stages of *Arabidopsis thaliana*. *Phytochemistry* 62 471–481. 10.1016/S0031-9422(02)00549-6 12620360

[B10] Burton R. L. (1969). *Mass Rearing the Corn Earworm in the Laboratory.* USDA ARS, Production Research Report No. 154. Washington, DC: United States Department of Agriculture.

[B11] Chow J. K. Akhtar Y. Isman M. B. (2005). The effects of larval experience with a complex plant latex on subsequent feeding and oviposition by the cabbage looper moth: *Trichoplusia ni* (Lepidoptera: Noctuidae). *Chemoecology* 15 129–133. 10.1007/s00049-005-0304-x

[B12] Clancy K. M. Price P. W. (1987). Rapid herbivore growth enhances enemy attack: Sublethal plant defenses remain a paradox. *Ecology* 68 733–737. 10.2307/1938479

[B13] Crawley M. J. (2013). *The R book.* Hoboken, NJ: John Wiley & Sons, Ltd.

[B14] Docimo T. Reichelt M. Schneider B. Kai M. Kunert G. Gershenzon J. (2012). The first step in the biosynthesis of cocaine in *Erythroxylum coca*: the characterization of arginine and ornithine decarboxylases. *Plant Mol. Biol.* 78 599–615. 10.1007/s11103-012-9886-1 22311164

[B15] Fahey J. W. Zalcmann A. T. Talalay P. (2001). The chemical diversity and distribution of glucosinolates and isothiocyanates among plants. *Phytochemistry* 56 5–51. 10.1016/S0031-9422(00)00316-2 11198818

[B16] Firempong S. Zalucki M. (1989). Host plant preferences of populations of *Helicoverpa armigera* (Hubner) (Lepidoptera, Noctuidae) from different geographic locations. *Aust. J. Zool.* 37 665–673. 10.1071/ZO9890665

[B17] Geu-Flores F. Olsen C. E. Halkier B. A. (2009). Towards engineering glucosinolates into non-cruciferous plants. *Planta* 229 261–270. 10.1007/s00425-008-0825-y 18830705

[B18] Hartmann T. (2007). From waste products to ecochemicals: fifty years research of plant secondary metabolism. *Phytochemistry* 68 2831–2846. 10.1016/j.phytochem.2007.09.017 17980895

[B19] Heckel D. G. (2014). “Insect detoxification and sequestration strategies,” in *Insect-Plant Interactions* Vol. 47 eds Voelckel C. Jander G. (Chichester: John Wiley & Sons, Ltd.) 77–114. 10.1002/9781118472507.ch3

[B20] Heidel-Fischer H. M. Vogel H. (2015). Molecular mechanisms of insect adaptation to plant secondary compounds. *Curr. Opin. Insect Sci.* 8 8–14. 10.1016/j.cois.2015.02.004 32846688

[B21] Hothorn T. Bretz F. Westfall P. (2008). Simultaneous inference in general parametric models. *Biom. J.* 50 346–363. 10.1002/bimj.200810425 18481363

[B22] Jeschke V. Gershenzon J. Vassão D. G. (2016a). “Insect detoxification of glucosinolates and their hydrolysis products,” in *Advances in Botanical Research* Vol. 80 ed. Kopriva S. (Amsterdam: Elsevier Ltd.) 199–245. 10.1016/bs.abr.2016.06.003

[B23] Jeschke V. Gershenzon J. Vassão D. G. (2016b). A mode of action of glucosinolate-derived isothiocyanates: detoxification depletes glutathione and cysteine levels with ramifications on protein metabolism in *Spodoptera littoralis*. *Insect Biochem. Mol. Biol.* 71 37–48. 10.1016/j.ibmb.2016.02.002 26855197

[B24] Kliebenstein D. J. Lambrix V. M. Reichelt M. Gershenzon J. Mitchell-Olds T. (2001). Gene duplication in the diversification of secondary metabolism: tandem 2-oxoglutarate–dependent dioxygenases control glucosinolate biosynthesis in Arabidopsis. *Plant Cell* 13 681–693. 10.1105/tpc.13.3.681 11251105 PMC135509

[B25] Lenth R. V. (2016). Least-squares means: The R package lsmeans. *J. Stat. Softw.* 69 1–33. 10.18637/jss.v069.i01

[B26] Li Q. Eigenbrode S. D. Stringham G. R. Thiagarajah M. R. (2000). Feeding and growth of *Plutella xylostella* and *Spodoptera eridania* on *Brassica juncea* with varying glucosinolate concentrations and myrosinase activities. *J. Chem. Ecol.* 26 2401–2419. 10.1023/A:1005535129399

[B27] Lichtenstein E. P. Morgan D. G. Strong F. M. (1962). Naturally occurring insecticides - Identification of 2-phenylethylisothiocyanate as an insecticide occuring naturally in edible part of turnips. *J. Agric. Food Chem.* 10 30–33. 10.1021/Jf60119a009

[B28] Malka O. Shekov A. Reichelt M. Gershenson J. Vassão D. G. Morin S. (2016). Glucosinolate desulfation by the phloem-feeding insect *Bemisia tabaci*. *J. Chem. Ecol.* 42 230–235. 10.1007/s10886-016-0675-1 26961756

[B29] Mithen R. Raybould A. F. Giamoustaris A. (1995). Divergent selection for secondary metabolites between wild populations of *Brassica oleracea* and its implications for plant-herbivore interactions. *Heredity* 75 472–484. 10.1038/Hdy.1995.164

[B30] Mosleh Arany A. de Jong T. J. Kim H. K. van Dam N. M. Choi Y. H. Verpoorte R. (2008). Glucosinolates and other metabolites in the leaves of *Arabidopsis thaliana* from natural populations and their effects on a generalist and a specialist herbivore. *Chemoecology* 18 65–71. 10.1007/s00049-007-0394-8

[B31] Müller R. de Vos M. Sun J. Y. Sønderby I. E. Halkier B. A. Wittstock U. (2010). Differential effects of indole and aliphatic glucosinolates on lepidopteran herbivores. *J. Chem. Ecol.* 36 905–913. 10.1007/s10886-010-9825-z 20617455

[B32] Pedras M. S. C. Nycholat C. M. Montaut S. Xu Y. M. Khan A. Q. (2002). Chemical defenses of crucifers: elicitation and metabolism of phytoalexins and indole-3-acetonitrile in brown mustard and turnip. *Phytochemistry* 59 611–625. 10.1016/S0031-9422(02)00026-2 11867093

[B33] Pentzold S. Zagrobelny M. Bjarnholt N. Kroymann J. Vogel H. Olsen C. E. (2015). Metabolism, excretion and avoidance of cyanogenic glucosides in insects with different feeding specialisations. *Insect Biochem. Mol. Biol.* 66 119–128. 10.1016/j.ibmb.2015.10.004 26483288

[B34] Pentzold S. Zagrobelny M. Roelsgaard P. S. Møller B. L. Bak S. (2014). The multiple strategies of an insect herbivore to overcome plant cyanogenic glucoside defence. *PLOS ONE* 9:e91337. 10.1371/journal.pone.0091337 24625698 PMC3953384

[B35] Perkins W. D. Jones R. L. Sparks A. N. Wiseman B. R. Snow J. W. McMillian W. W. (1973). *Artificial diets for mass rearing the corn earworm (Heliothis zea).* Washington, DC: U.S. Department of Agriculture.

[B36] Pinheiro J. Bates D. DebRoy S. Sarkar D. R Core Team (2017). *nlme: Linear and Nonlinear Mixed Effects Models_. R package version 3.1-131*. Available at: https://CRAN.R-project.org/package=nlme

[B37] Popova T. A. (1993). A study of antibiotic effects of cabbage cultivars on the cabbage moth *Mamestra brassicae* L. (Lepidoptera, Noctuidae). *Entomol. Rev.* 72 125–132.

[B38] Proffit M. Khallaf M. A. Carrasco D. Larsson M. C. Anderson P. (2015). ‘Do you remember the first time?’ Host plant preference in a moth is modulated by experiences during larval feeding and adult mating. *Ecol. Lett.* 18 365–374. 10.1111/ele.12419 25735877

[B39] R Core Team (2017). *R: A Language and Environment for Statistical Computing.* Vienna: R Foundation for Statistical Computing.

[B40] Ratzka A. Vogel H. Kliebenstein D. J. Mitchell-Olds T. Kroymann J. (2002). Disarming the mustard oil bomb. *Proc. Natl. Acad. Sci. U.S.A.* 99 11223–11228. 10.1073/pnas.172112899 12161563 PMC123237

[B41] Santolamazza-Carbone S. Sotelo T. Velasco P. Cartea M. E. (2015). Antibiotic properties of the glucosinolates of *Brassica oleracea* var. acephala similarly affect generalist and specialist larvae of two lepidopteran pests. *J. Pest Sci.* 89 195–206. 10.1007/s10340-015-0658-y

[B42] Schlaeppi K. Bodenhausen N. Buchala A. Mauch F. Reymond P. (2008). The glutathione-deficient mutant pad2-1 accumulates lower amounts of glucosinolates and is more susceptible to the insect herbivore *Spodoptera littoralis*. *Plant J.* 55 774–786. 10.1111/j.1365-313X.2008.03545.x 18466300

[B43] Schramm K. Vassão D. G. Reichelt M. Gershenzon J. Wittstock U. (2012). Metabolism of glucosinolate-derived isothiocyanates to glutathione conjugates in generalist lepidopteran herbivores. *Insect Biochem. Mol. Biol.* 42 174–182. 10.1016/j.ibmb.2011.12.002 22193392

[B44] Sønderby I. E. Geu-Flores F. Halkier B. A. (2010). Biosynthesis of glucosinolates - Gene discovery and beyond. *Trends Plant Sci.* 15 283–290. 10.1016/j.tplants.2010.02.005 20303821

[B45] Sønderby I. E. Hansen B. G. Bjarnholt N. Ticconi C. Halkier B. A. Kliebenstein D. J. (2007). A systems biology approach identifies a R2R3 MYB gene subfamily with distinct and overlapping functions in regulation of aliphatic glucosinolates. *PLOS ONE* 2:e1322. 10.1371/journal.pone.0001322 18094747 PMC2147653

[B46] Sun J. Y. Sønderby I. E. Halkier B. A. Jander G. de Vos M. (2009). Non-volatile intact indole glucosinolates are host recognition cues for ovipositing *Plutella xylostella*. *J. Chem. Ecol.* 35 1427–1436. 10.1007/s10886-009-9723-4 20054620

[B47] Textor S. Gershenzon J. (2009). Herbivore induction of the glucosinolate-myrosinase defense system: major trends, biochemical bases and ecological significance. *Phytochem. Rev.* 8 149–170. 10.1007/s11101-008-9117-1

[B48] Therneau T. (2015). *A Package for Survival Analysis in S. version 2.38.* Available at: http://CRAN.R-project.org/package=survival

[B49] Thöming G. Larsson M. C. Hansson B. S. Anderson P. (2013). Comparison of plant preference hierarchies of male and female moths and the impact of larval rearing hosts. *Ecology* 94 1744–1752. 10.1890/12-0907.1 24015518

[B50] Wittstock U. Agerbirk N. Stauber E. J. Olsen C. E. Hippler M. Mitchell-Olds T. (2004). Successful herbivore attack due to metabolic diversion of a plant chemical defense. *Proc. Natl. Acad. Sci. U.S.A.* 101 4859–4864. 10.1073/pnas.0308007101 15051878 PMC387339

[B51] Wittstock U. Burow M. (2010). Glucosinolate breakdown in Arabidopsis: mechanism, regulation and biological significance. *Arabidopsis Book* 8:e0134. 10.1199/tab.0134 22303260 PMC3244901

[B52] Wittstock U. Gershenzon J. (2002). Constitutive plant toxins and their role in defense against herbivores and pathogens. *Curr. Opin. Plant Biol.* 5 300–307. 10.1016/S1369-5266(02)00264-9 12179963

[B53] Zhao Y. Hull A. K. Gupta N. R. Goss K. A. Alonso J. Ecker J. R. (2002). Trp-dependent auxin biosynthesis in Arabidopsis: involvement of cytochrome P450s CYP79B2 and CYP79B3. *Genes Dev.* 16 3100–3112. 10.1101/gad.1035402 12464638 PMC187496

[B54] Zou X. Xu Z. Zou H. Liu J. Chen S. Feng Q. (2016). Glutathione *S*-transferase SlGSTE1 in *Spodoptera litura* may be associated with feeding adaptation of host plants. *Insect Biochem. Mol. Biol.* 70 32–43. 10.1016/j.ibmb.2015.10.005 26631599

[B55] Zuur A. F. Ieno E. N. Walker N. Saveliev A. A. Smith G. M. (2009). *Mixed Effects Models and Extensions in Ecology with R.* New York, NY: Springer 101–142.

