# Supplementary material for: How Glucosinolates Affect Generalist Lepidopteran Larvae: Growth, Development and Glucosinolate Metabolism
Source: Front Plant Sci. 2017 Nov 21;8:1995. doi: 10.3389/fpls.2017.01995 (PMC5702293; doi:10.3389/fpls.2017.01995)
Supplement: Supplementary file 1 [file Table_1.docx]

**Supplementary Table S1.** **Metabolite concentrations in seven-week-old freeze-dried whole *A. thaliana* plants (aerial parts)** used in experiment to assess insect development. Listed are the mean ± standard error (N=7) and statistical testing was performed using ANOVA. DW: dry weight; GLS: glucosinolate. For explanation of abbreviations for individual glucosinolates, see the legend of Fig. 2.

|  |  | **wild type** | **aliphatic GLS only** | **indolic GLS**  **only** | **no GLS** | ***P* value** | ***F* value** |
| --- | --- | --- | --- | --- | --- | --- | --- |
| **GLS** | **total GLS** | 21.29 ± 2.31 | 17.19 ± 1.60 | 3.44 ± 0.36 | 0.01 ± 0.00 |  |  |
| **[µmol/g DW]** | **total aliphatic** | 19.12 ± 2.09 | 17.17 ± 1.60 | 0.00 | 0.00 |  |  |
|  | **total indolic** | 2.16 ± 0.23 | 0.01 ± 0.00 | 3.44 ± 0.36 | 0.01 ± 0.00 |  |  |
|  | **3MSOP** | 1.84 ± 0.19 | 1.48 ± 0.14 | 0.00 | 0.00 |  |  |
|  | **4MSOB** | 14.19 ± 1.58 | 12.41 ± 1.15 | 0.00 | 0.00 |  |  |
|  | **5MSOP** | 0.39 ± 0.04 | 0.40 ± 0.03 | 0.00 | 0.00 |  |  |
|  | **7MSOH** | 0.24 ± 0.03 | 0.31 ± 0.04 | 0.00 | 0.00 |  |  |
|  | **4MTB** | 1.14 ± 0.14 | 1.08 ± 0.11 | 0.00 | 0.00 |  |  |
|  | **8MSOO** | 1.32 ± 0.15 | 1.49 ± 0.18 | 0.00 | 0.00 |  |  |
|  | **I3M** | 1.39 ± 0.16 | 0.01 ± 0.00 | 2.63 ± 0.27 | 0.01 ± 0.00 |  |  |
|  | **4MOI3M** | 0.66 ± 0.08 | 0.00 | 0.62 ± 0.10 | 0.00 |  |  |
|  | **1MOI3M)** | 0.12 ± 0.01 | 0.00 | 0.19 ± 0.01 | 0.00 |  |  |
| **carbohydrates** | **sucrose** | 1.53 ± 0.23 | 1.83 ± 0.15 | 1.71 ± 0.21 | 1.87 ± 0.22 | 0.706 | 0.471 |
| **[µmol/g DW]** | **fructose** | 0.66 ± 0.10 | 0.79 ± 0.06 | 1.02 ± 0.17 | 0.65 ± 0.10 | 0.150 | 1.944 |
|  | **glucose + galactose** | 1.31 ± 0.30 | 1.15 ± 0.11 | 2.78 ± 0.67 | 1.15 ± 0.11 | 0.023 | 3.818 |
| **amino acids**  **[µmol/g DW]** |  | 161.09 ± 16.54 | 165.16 ± 17.05 | 150.55 ± 22.70 | 177.47 ± 15.67 | 0.811 | 0.320 |
| **soluble protein**  **[% dry weight]** |  | 1.22 ± 0.15 | 1.38 ± 0.14 | 1.40 ± 0.14 | 1.29 ± 0.12 | 0.503 | 0.806 |
