# Supplementary material for: How Glucosinolates Affect Generalist Lepidopteran Larvae: Growth, Development and Glucosinolate Metabolism
Source: Front Plant Sci. 2017 Nov 21;8:1995. doi: 10.3389/fpls.2017.01995 (PMC5702293; doi:10.3389/fpls.2017.01995)
Supplement: Supplementary file 2 [file Table_2.docx]

**Supplementary Table S2.** **Larval survival at different developmental stages**. Percentages of larvae that survived until day 6 (end of group phase; 100% = 10, *N* = 10), until the 6^th^ instar (end of individual phase; 100% = 18) and successfully pupated (100% = number of 6^th^ instar larvae). Statistical testing was performed with an *ANOVA* for % survival day 6, and *proportion test* for % survival to 6^th^ instar and % pupation*.* GLS: glucosinolate.

|  |  | **wild type** | **aliphatic**  **GLS only** | **indolic**  **GLS only** | **no**  **GLS** | ***P* value** | ***F/ ^ ^***  **value** |
| --- | --- | --- | --- | --- | --- | --- | --- |
| ***Spodoptera littoralis*** | % survival day 6 (early development) | 87 ± 3 | 91 ± 3 | 90 ± 3 | 94 ± 2 | n.s. | 1.095 |
|  | % survival to 6^th^ instar (late development) | 89 | 89 | 78 | 89 | n.s. | 1.394 |
|  | % pupation | 88 | 81 | 79 | 100 | n.s. | 3.765 |
| ***Mamestra brassicae*** | % survival day 6 (early development) | 91 ± 3 | 94 ± 3 | 92 ± 2 | 94 ± 2 | n.s. | 0.276 |
|  | % survival to 6^th^ instar (late development) | 100 | 100 | 100 | 94 | n.s. | 3.042 |
|  | % pupation | 100 | 94 | 100 | 94 | n.s. | 2.120 |
