# Supplementary material for: How Glucosinolates Affect Generalist Lepidopteran Larvae: Growth, Development and Glucosinolate Metabolism
Source: Front Plant Sci. 2017 Nov 21;8:1995. doi: 10.3389/fpls.2017.01995 (PMC5702293; doi:10.3389/fpls.2017.01995)
Supplement: Supplementary file 3 [file Table_3.docx]

**Supplementary Table S3. Average larval weights (in mg) at different developmental stages**: after early development (beginning of the 3^rd^ instar, day 6 for *S. littoralis* and day 10 for *M. brassicae*) and after late development (beginning of the 6^th^ instar, day 20 for *S. littoralis* and day 28 for *M. brassicae*). Listed is the mean ± standard error in mg. Statistical analysis was performed with an *ANOVA*, and the letters denote significantly different groups based on a Tukey post hoc test (0.05 level). GLS: glucosinolate.

|  |  | **wild type** | **aliphatic**  **GLS only** | **indolic**  **GLS only** | **no GLS** | ***P* value** | ***F* value** |
| --- | --- | --- | --- | --- | --- | --- | --- |
|  |  |  |  |  |  |  |  |
| *Spodoptera*  *littoralis* | day 6 | 1.85 ± 0.13 (a) | 2.56 ± 0.17 (b) | 4.65 ± 0.31 (c) | 5.44 ± 0.42 (c) | < 0.001 | 45.247 |
|  | day 20 | 204.70 ± 17.32 (a) | 437.83 ± 48.63 (b) | 571.71 ± 51.52 (b) | 765.50 ± 32.85 (c) | < 0.001 | 103.466 |
| *Mamestra*  *brassicae* | day 10 | 1.63 ± 0.15 (a) | 2.31 ± 0.19 (b) | 3.78 ± 0.29 (c) | 3.73 ± 0.19 (c) | < 0.001 | 57.083 |
|  | day 28 | 199.83 ± 22.97 (a) | 516.18 ± 29.42 (b) | 568.74 ± 29.82 (b) | 711.64 ± 29.09 (c) | < 0.001 | 104.937 |
