# Supplementary material for: How Glucosinolates Affect Generalist Lepidopteran Larvae: Growth, Development and Glucosinolate Metabolism
Source: Front Plant Sci. 2017 Nov 21;8:1995. doi: 10.3389/fpls.2017.01995 (PMC5702293; doi:10.3389/fpls.2017.01995)
Supplement: Supplementary file 4 [file Table_4.docx]

**Supplementary Table S4.** **Average times of instar changes and pupation**, in days during the two phases of the development experiment: 1^st^-3^rd^ instar (larvae measured in groups) and 4^th^ instar-pupa (larvae measured as individuals). Listed is the mean ± standard error. Statistical significance was analyzed with *ANOVA*, and the letters denote significantly different groups based on a Tukey post hoc test (0.05 level). For details on the calculation and statistics, see Materials and Methods. GLS: glucosinolates.

|  |  | **instar change to** | **wild type (WT)** | **aliphatic**  **GLS only** | **indolic**  **GLS only** | **no GLS** | ***P***  **value** | ***F* value** | **Δ WT- no GLS**  **(cumulative)** |
| --- | --- | --- | --- | --- | --- | --- | --- | --- | --- |
| *Spodoptera littoralis* | group  phase | 2^nd^ | 4.39 ± 0.14 (a) | 3.94 ± 0.05  (b) | 3.24 ± 0.10 (c) | 3.04 ± 0.12 (c) | <0.001 | 33.183 | 1.4 |
|  |  | 3^rd^ | 7.32 ± 0.17 (a) | 6.84 ± 0.22  (b) | 5.89 ± 0.06 (c) | 5.54 ± 0.13 (c) | <0.001 | 51.333 | 1.8 |
|  | individual  phase | 4^th^ | 13.14 ± 0.05 (a) | 12.24 ± 0.18 (b) | 11.03 ± 0.12 (c) | 10.47 ± 0.07 (d) | <0.001 | 104.988 | 2.7 |
|  |  | 5^th^ | 16.70 ± 0.12 (a) | 15.35 ± 0.10 (b) | 14.12 ± 0.04 (c) | 13.61 ± 0.06 (d) | <0.001 | 258.278 | 3.1 |
|  |  | 6^th^ | 20.46 ± 0.22 (a) | 18.89 ± 0.03 (b) | 17.32 ± 0.08 (c) | 16.35 ± 0.48 (c) | <0.001 | 44.926 | 4.1 |
|  |  | pupation | 25.60 ± 0.35 (a) | 24.79 ± 0.10 (b) | 22.89 ± 0.15 (c) | 21.4 ± 0.09 (d) | <0.001 | 70.563 | 3.7 |
| *Mamestra brassicae* | group  phase | 2^nd^ | 3.80 ± 0.19 (a) | 3.16 ± 0.07  (b) | 2.91 ± 0.02 (c) | 2.85 ± 0.02 (c) | <0.001 | 17.315 | 0.9 |
|  |  | 3^rd^ | 8.25 ± 0.17 (a) | 7.32 ± 0.26  (b) | 6.22 ± 0.35 (c) | 6.14 ± 0.21 (c) | <0.001 | 74.717 | 2.1 |
|  | individual  phase | 4^th^ | 15.10 ± 0.13 (a) | 11.23 ± 1 0.11 (b) | 9.80 ± 0.46 (c) | 9.88 ± 0.07 (c) | <0.001 | 101.304 | 5.2 |
|  |  | 5^th^ | 20.99 ± 0.14 (a) | 16.68 ± 0.10 (b) | 15.12 ± 0.23 (c) | 14.32 ± 0.23 (d) | <0.001 | 268.809 | 6.7 |
|  |  | 6^th^ | 28.58 ± 0.19 (a) | 22.81 ± 0.11 (b) | 22.13 ± 0.08 (c) | 21.50 ± 0.03 (d) | <0.001 | 757.751 | 7.1 |
|  |  | pupation | 36.41 ± 0.17 (a) | 34.41 ± 0.14 (b) | 32.05 ± 0.08 (c) | 31.65 ± 0.46 (c) | <0.001 | 73.676 | 4.8 |
