# Supplementary material for: How Glucosinolates Affect Generalist Lepidopteran Larvae: Growth, Development and Glucosinolate Metabolism
Source: Front Plant Sci. 2017 Nov 21;8:1995. doi: 10.3389/fpls.2017.01995 (PMC5702293; doi:10.3389/fpls.2017.01995)
Supplement: Supplementary file 5 [file Table_5.docx]

**Supplementary Table S5. Larval weights directly before instar changes**. Data are presented as the estimates of the means ± standard error in mg fresh weight from the generalized least squares model. The *P* values were calculated with an *ANOVA* and the letters denote significantly different groups based on a *Tukey post-hoc test* (0.05 level). For details on the calculation and statistics, see Materials and Methods. GLS: glucosinolate.

|  | **weight before the change to** | **wild type** | **aliphatic**  **GLS only** | **indolic**  **GLS only** | **no GLS** | ***P* value** | ***F* value** |
| --- | --- | --- | --- | --- | --- | --- | --- |
|  |  |  |  |  |  |  |  |
| *Spodoptera littoralis* | 4^th^ instar | 10.58 ± 0.76 | 10.77 ± 0.47 | 10.63 ± 0.90 | 10.47 ± 0.98 | n.s. | 0.025 |
|  | 5^th^ instar | 52.67 ± 2.45 | 50.86 ± 3.08 | 50.17 ± +6.58 | 48.84 ± 3.54 | n.s. | 0.161 |
|  | 6^th^ instar | 250.01 ± 15.23 | 234.86 ± 9.70 | 202.92 ± 13.34 | 235.03 ± 22.68 | n.s. | 1.353 |
| *Mamestra brassicae* | 4^th^ instar | 8.88 ± 0.55 (a) | 10.84 ± 0.33 (b) | 10.51 ± 0.28 (b) | 10.87 ± 0.57 (b) | 0.008 | 4.413 |
|  | 5^th^ instar | 39.46 ± 2.50 (a) | 47.74 ± 1.83 (b) | 43.11 ± 1.75 (a) | 43.48 ± 1.44 (a) | 0.031 | 3.157 |
|  | 6^th^ instar | 223.13 ± 15.46 | 218.03 ± 7.45 | 224.44 ± 8.50 | 221.10 ± 7.46 | n.s. | 0.093 |
