# Supplementary material for: How Glucosinolates Affect Generalist Lepidopteran Larvae: Growth, Development and Glucosinolate Metabolism
Source: Front Plant Sci. 2017 Nov 21;8:1995. doi: 10.3389/fpls.2017.01995 (PMC5702293; doi:10.3389/fpls.2017.01995)
Supplement: Supplementary file 6 [file Table_6.docx]

**Supplementary Table S6**. **Statistical analysis of the total amount of 4msob-ITC conjugates (pmol/mg dry feces) excreted by larvae at different stages of development**. For details of the analyses, refer to Materials and Methods. WT: wild type Col-0, Cyp: plant line with aliphatic GLSs

| **species** | **age/set-up** | **transformation** | **explanatory variable** | **likelihood ratio** | ***P* value** | **posthoc** |
| --- | --- | --- | --- | --- | --- | --- |
| *Spodoptera littoralis* | early/group | log | day | 11.55 | 0.003 | day3 = day5 > day7 |
|  |  |  | plant | 0.10 | n.s. |  |
|  |  |  | day*plant interaction | 16.31 | <0.001 | Cyp5 > Cyp3 = WT3 = WT5 = WT7 > Cyp7 |
|  | late/individual | log | instar | 22.75 | <0.001 | L3=L4>L5=L6 |
|  |  |  | plant | 0.96 | n.s. |  |
|  |  |  | instar*plant interaction | 0.20 | n.s. |  |
| *Mamestra brassicae* | early/group | log | day | 11.70 | 0.020 | day3=day5=day7=day9<day10 |
|  |  |  | plant | 2.58 | n.s. |  |
|  |  |  | day*plant interaction | 34.72 | <0.001 | Cyp3+7+9+10+WT3+5 < Cyp5+WT7+9 < WT10 |
|  | late/individual | log | instar | 1.52 | n.s. |  |
|  |  |  | plant | 4.26 | n.s. |  |
|  |  |  | instar*plant interaction | 3.13 | n.s. |  |
