# Supplementary material for: How Glucosinolates Affect Generalist Lepidopteran Larvae: Growth, Development and Glucosinolate Metabolism
Source: Front Plant Sci. 2017 Nov 21;8:1995. doi: 10.3389/fpls.2017.01995 (PMC5702293; doi:10.3389/fpls.2017.01995)
Supplement: Supplementary file 7 [file Table_7.docx]

**Supplementary Table S7.** **Statistical analysis of the relative proportions of each individual 4msob-ITC conjugate in feces.** For details of the analysis, refer to Materials and Methods. WT: wild type Col-0, Cyp: plant line with aliphatic GLSs only, numbers (e.g. WT5) refer to the day of sampling

| **species** | **age/**  **set-up** | **conjugate**  **% of total** | **trans-formation** | **explanatory variable** | **likelihood ratio** | ***P* value** | **posthoc** |
| --- | --- | --- | --- | --- | --- | --- | --- |
| *Spodoptera littoralis* | early/group | 4msob-GSH | arcsin sqrt | day | 2.911 | n.s. |  |
|  |  |  |  | plant | 0.474 | n.s. |  |
|  |  |  |  | day*plant interaction | 3.752 | n.s. |  |
|  |  | 4msob-CysGly | arcsin sqrt | day | 8.909 | 0.012 | day3>day5=day7 |
|  |  |  |  | plant | 0.107 | n.s. |  |
|  |  |  |  | day*plant interaction | 7.051 | 0.029 | Cyp3>Cyp5=Cyp7=WT3=WT5=WT7 |
|  |  | 4msob-Cys | log | day | 20.383 | <0.001 | day3<day5=day7 |
|  |  |  |  | plant | 6.804 | 0.009 | Cyp>WT |
|  |  |  |  | day*plant interaction | 3.891 | n.s. |  |
|  |  | 4msob-NAC | log | day | 9.349 | 0.009 | day3<day5=day7 |
|  |  |  |  | plant | 0.533 | n.s. |  |
|  |  |  |  | day*plant interaction | 1.889 | n.s. |  |
|  | late/individual | 4msob-GSH | log | instar | 13.444 | 0.004 | L3=L5=L6<L4 |
|  |  |  |  | plant | 0.834 | n.s. |  |
|  |  |  |  | instar*plant interaction | 3.289 | n.s. |  |
|  |  | 4msob-CysGly | arcsin sqrt | instar | 35.899 | <0.001 | L3>L4>L5=L6 |
|  |  |  |  | plant | 1.699 | n.s. |  |
|  |  |  |  | instar*plant interaction | 2.477 | n.s. |  |
|  |  | 4msob-Cys | arcsin sqrt | instar | 30.696 | <0.001 | L3=L4<L5=L6 |
|  |  |  |  | plant | 0.458 | n.s. |  |
|  |  |  |  | instar*plant interaction | 4.277 | n.s. |  |
|  |  | 4msob-NAC | log | instar | 75.154 | <0.001 | L3=L4<L5<L6 |
|  |  |  |  | plant | 3.817 | 0.051 | WT > Cyp |
|  |  |  |  | instar*plant interaction | 0.529 | n.s. |  |

| **species** | **age/**  **set-up** | **conjugate**  **% of total** | **trans-formation** | **explanatory variable** | **likelihood ratio** | *P* **value** | **posthoc** |
| --- | --- | --- | --- | --- | --- | --- | --- |
| *Mamestra brassicae* | early/group | 4msob-GSH | log | day | 18.381 | 0.001 | day3<day5=day9=day10<day7 |
|  |  |  |  | plant | 1.636 | n.s. |  |
|  |  |  |  | day*plant interaction | 9.251 | n.s. |  |
|  |  | 4msob-CysGly | arcsin sqrt | day | 22.597 | <0.001 | day3=day5 > day7=day9=day10 |
|  |  |  |  | plant | 7.535 | 0.006 | Cyp > WT |
|  |  |  |  | day*plant interaction | 24.996 | <0.001 | Cyp7+9+10 < Cyp5+WT3+5+7+9+10 < Cyp3 |
|  |  | 4msob-Cys | log | day | 0.796 | n.s. |  |
|  |  |  |  | plant | 5.309 | 0.021 | Cyp > WT |
|  |  |  |  | day*plant interaction | 21.428 | <0.001 | WT10 < Cyp3=Cyp5=WT7=WT9 < Cyp7=Cyp9=Cyp10=WT3=WT5 |
|  |  | 4msob-NAC | log | day | 20.961 | <0.001 | day3=day5=day7 < day9=day10 |
|  |  |  |  | plant | 4.103 | 0.043 | Cyp > WT |
|  |  |  |  | day*plant interaction | 16.949 | 0.002 | Cyp3=Cyp7=WT3=WT5=WT7=WT9 < Cyp5=Cyp10=WT10 < Cyp9 |
|  | late/individual | 4msob-GSH | log | instar | 103.383 | <0.001 | L4 > L5 > L6 |
|  |  |  |  | plant | 5.498 | 0.019 | WT > Cyp |
|  |  |  |  | instar*plant interaction | 16.612 | <0.001 | Cyp4=WT4 > Cyp5=WT5 > WT6 > Cyp6 |
|  |  | 4msob-CysGly | log | instar | 26.630 | <0.001 | L4 = L5 > L6 |
|  |  |  |  | plant | 2.780 | n.s. |  |
|  |  |  |  | instar*plant interaction | 1.808 | n.s. |  |
|  |  | 4msob-Cys | arcsin sqrt | instar | 48.209 | <0.001 | L4 < L5 < L6 |
|  |  |  |  | plant | 1.161 | n.s. |  |
|  |  |  |  | instar*plant interaction | 2.035 | n.s. |  |
|  |  | 4msob-NAC | log | instar | 23.127 | <0.001 | L4 < L5 < L6 |
|  |  |  |  | plant | 0.283 | n.s. |  |
|  |  |  |  | instar*plant interaction | 0.210 | n.s. |  |
