# Supplementary material for: How Glucosinolates Affect Generalist Lepidopteran Larvae: Growth, Development and Glucosinolate Metabolism
Source: Front Plant Sci. 2017 Nov 21;8:1995. doi: 10.3389/fpls.2017.01995 (PMC5702293; doi:10.3389/fpls.2017.01995)
Supplement: Supplementary file 8 [file Table_8.docx]

**Supplementary Table S8. Comparison of leaf fragment sizes in feces from 4^th^ and 6^th^ instar *S. littoralis* caterpillars fed on *A. thaliana* Col-0 WT or the no-GLS plant line**. Five caterpillars were allowed to feed for 24 h on each plant line. Feces were collected and resuspended in water, and three microscopy slides were prepared per caterpillar. For each slide, the pictures of five areas were recorded and the perimeters of all fragments in each picture were obtained. All perimeters derived from each caterpillar were averaged, and the data presented as the mean ± SE. Statistical testing was performed with a *two-way ANOVA* (plant: *P* = n.s., *F* = 0.130; instar: *P* = n.s., *F* = 0.202; plant:instar: *P* = n.s.; *F* = 0.943).

| **Leaf fragment perimenter [mm]** | **wild type** | **no GLS** |
| --- | --- | --- |
| 4^th^ instar | 1.08 ± 0.10 | 1.05 ± 0.06 |
| 6^th^ instar | 1.18 ± 0.06 | 1.16 ± 0.07 |
