# Supplementary material for: How Glucosinolates Affect Generalist Lepidopteran Larvae: Growth, Development and Glucosinolate Metabolism
Source: Front Plant Sci. 2017 Nov 21;8:1995. doi: 10.3389/fpls.2017.01995 (PMC5702293; doi:10.3389/fpls.2017.01995)
Supplement: Supplementary file 9 [file Image_1.pdf]

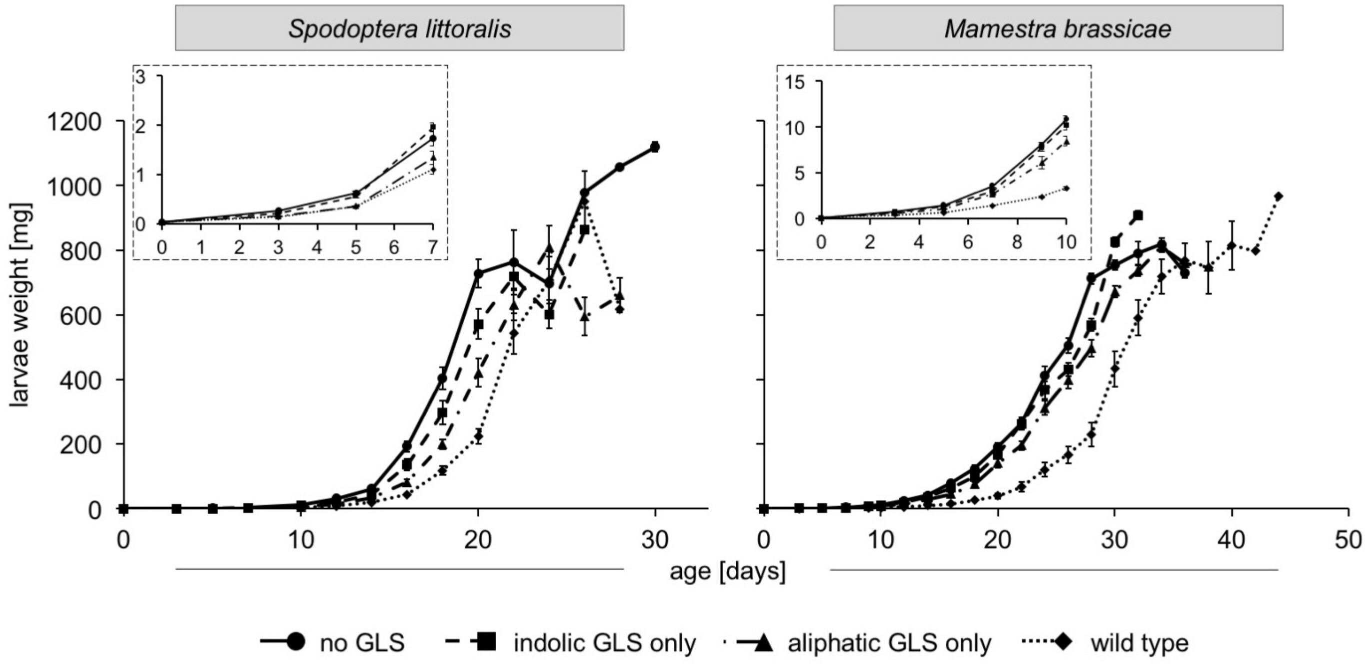

Supplementary Figure S1. Growth curves from hatching to pupation for *S. littoralis* and *M. brassicae.* Insert: Magnification during the early phase of the experiment (prior to the 3rd instar) when caterpillars were weighed in groups; in the later phase caterpillars were weighed as individuals. The data are presented as mean ± standard error. GLS: glucosinolates
